# The Association of Menstruation and Leisure-Time Physical Activity among Korean Female University Students: A Preliminary Study

**DOI:** 10.3390/ijerph19127492

**Published:** 2022-06-18

**Authors:** Bo-Ram Kim, Sunghwun Kang, Woo-Suk Jeong

**Affiliations:** 1 Department of Physical Education, College of Education, Korea University, Seoul 02841, Korea; brh@naver.com; 2 Laboratory of Exercise Physiology, Department of Sport Science, Kangwon National University, Chuncheon 24341, Korea; 94psycho@kangwon.ac.kr; 3 Interdisciplinary Program in Biohealth-Machinery Convergence Engineering, Kangwon National University, Chuncheon 24341, Korea; 4 Laboratory of Sport Sociology, Department of Marine Sports, Pukyong National University, Busan 48513, Korea

**Keywords:** health consciousness (HC), leisure-time physical activity (LTPA), menstruation, amount of LTPA, intention to LTPA

## Abstract

An active lifestyle elicits many health benefits. This study’s aim is to understand the actual of leisure time physical activity (LTPA) of female university students in Korea who are experiencing stress due to, e.g., study and employment. LTPA is the degree of exercise participation in leisure time; it is cardiovascular and strength-based behavior occurring within recreation, exercise or sport and provides a positive effect on life satisfaction and psychological well-being. LTPA has been recommended as a method of reducing menstrual symptom severity. However, a lack of evidence exists to support a clear relationship between LTPA and menstruation in female university students. Health consciousness (HC) refers to the degree of interest in one’s health, and Korean female university students pay considerable attention to their body shape, diet habits, and LTPA. This study investigated female university students (K University in Gangwon-do, D University in Busan, and U University in Ulsan) in three metropolitan cities in Korea. Specifically, after seeking cooperation from the person in charge of each university, the purpose of this study was explained. In addition, after promising to provide coffee coupons to students who wish to respond to the survey, cooperation was sought in recruiting research subjects through the person in charge of each university. In addition, a total of 36 surveys that did not have contents filled in fully or gave inconsistent answers were excluded among all survey participants. Chi-square test, *t*-test and univariate one-way ANOVA, and Pearson’s correlation coefficient analysis were performed for between groups (HC, LTPA, intention to participate in LTPA). The LTPA results in relation to the menstruation patterns of Korean female college students are shown. First, there were no significant differences between menstruation (regular vs. irregular) and general LTPA during non-menstrual time periods (χ^2^ = 5.828, *p* < 0.212). However, female university students with regular menstruation patterns had higher LTPA after menstruation compared to female university students with irregular menstruation. Second, there were significant differences in the relationships among HC (*p* = 0.000), amount of LTPA (*p* = 0.002), and intention to perform LTPA (*p* = 0.002), according to the menstruation patterns (regular vs. irregular) of Korean female university students. In other words, those with regular menstruation patterns showed higher HC, amount of LTPA and intention to participate in LTPA than those with irregular menstruations. Third, there were significant differences in the relationship among HC (*p* = 0.000), amount of LTPA (*p* = 0.000), and intention to participate in LTPA (*p* = 0.000) according to LTPA of Korean female university students. Causation cannot be inferred from correlational studies. Therefore, female university students ultimately have different HC and participation in LTPA depending on menstruation regularity.

## 1. Introduction

Physical activity is a broad term that encompasses both leisure time activity (sports, exercise) and activities of daily living (household living tasks, transportation) [1]. Leisure-time physical activity (LTPA) is cardiovascular and strength-based behavior occurring within recreation, exercise or sport [2]; in general, it is applied in various studies as the degree of participation in exercise in leisure time [3,4]. LTPA has been positively linked to life satisfaction and psychological well-being [5,6], and the importance of LTPA is emphasized through various studies performed previously such as BMI changes and LTPA [3], the relationship between LTPA and stress [7], and the relationship between LTPA and health-related quality of life [8]. Health consciousness (HC) refers to concern for health into daily life [9]. In other words, if one’s health consciousness is high, they more actively pursue healthy behaviors. Additionally, intention to participate in LTPA refers to the will to continue LTPA with positive psychological effects such as fun and pleasure. On the other hand, the LTPA participation rate of all citizens tended to decrease slightly in 2020 (60.1%) and 2021 (60.8%) compared to 2019 (66.6%) before the outbreak of COVID-19 [10].

Society in the 21st century can easily obtain information about people’s exercise and diet through various media. Particularly, university students who are interested in their appearance will try to manage their body shape with somewhat more vigorous effort. Additionally, some university students often experience physical and mental issues because of excessive effort to control weight [11]. A university student generally goes through a period of becoming an adult from adolescence and is prone to health issues because of unhealthy habits and behaviors [12]. Korean university students are exposed to psychological and social stresses that threaten their health (smoking, drinking, irregular eating habits, lack of sleep, etc.) as well as to a competitive social atmosphere, such as staying on top of the class, relationship issues, and worries about employment and career. They develop irregular lifestyles because of sudden changes in their lives, being given freedom and independence that they did not experience before, resulting in an irregular pattern in daily life which leads to some health issues [13]. In other words, most Korean university students are freed from the pressure they had during their high-school days. They are free from social expectations that burdened them while they were in high school, and this sudden freedom often leads them to form irregular lifestyles while not recognizing the importance of their physical and mental health [14]. Another problem is the slender bodies of celebrities, models, and sports stars appearing in various media, which affects female university students; the culture of distorted appearance is threatening health [15]. In other words, in modern society, the social ideal presented by mass media becomes a reference point. Korean university students go through excessive diet routines to maintain their body shape to seek employment after their graduation [16]. Unfortunately, this is expected by society, and these students go through excessive diet routines even though they know it is not good for their health [17,18]. Additionally, Korean female university students pay great attention to their body shape, diet habits, and LTPA. Their self-awareness of their health leads to this behavior. As such, Korean female university students have a high interest in health and physical activity [19]. Therefore, in order to maintain and improve exercise capacity, regular physical activity of at least 3 times a week and at least 30 min at a time is recommended [20].

One obstacle that these students face while trying to maintain their health is menstruation (periodic bleeding from the uterus in women). Women’s menstrual cycles differ from person to person, but occur, on average, every 28 days [21]. A cycle is typically as follows: menstrual phase, 1–5 days; follicular phase, 1–13 days; ovulation, 14 days; luteal phase, 14–28 days [22]. Estrogen and progesterone generated during menstruation cause physical changes such as heart rate, vasodilation, and muscle contractility, and psychological changes such as frustration, anger, anxiety, and depression [23]. Menstruation can present complexity in achieving the healthy body and mind that these female students wish to have. Female university students are stressed and sensitive due to various factors (study, career, employment, physical health, etc.) [24,25]. According to the 2021 National Sports Survey [10], the participation rate of LTPA was 61.4% for women, who responded that they participated ‘more than once a week’. In addition, women in their 20s were surveyed with a 60.7% participation rate. Specifically, the participation rate of women in their 20s was higher than that of women in their teens (49.8%) and 30s (55.7%), while it was lower than that of women in their 40s (70.4%). This study is to analyze the differences in HC, amount of LTPA, and intent to participate in LTPA according to the menstruation pattern (regular and irregular) and LTPA of Korean female university students. LTPA was defined operationally as follows: do not participate in LTPA, before menstruation (luteal phase), after menstruation (period excluding menstruation in the follicular phase), when not menstruating (any period without menstruation phase), always. Therefore, this study’s purpose is to understand the real situation of leisure time physical activity participation of female university students in Korea who are experiencing stress due to, e.g., study and employment.

## 2. Materials and Methods

### 2.1. Sampling Method

Korea had various restrictions due to a lot of confusion and COVID-19. In fact, 28.4% of the respondents said that LTPA has decreased in the past year in 2021, up 24.5% from 2019 before COVID-19 and 9.4% from 2020. In addition, 20.6% of the reasons for the decrease were concerned about COVID-19 infction [26]. Therefore, the convenience sampling method was inevitably used because there were many restrictions on the sampling of the study subjects. In addition, the survey contents of this study include sensitive and private contents such as menstrual cycles and menstrual medication. This study surveyed 500 students attending K University in Gangwon-do, D University in Busan, and U University in Ulsan. Specifically, after seeking cooperation from the person in charge of each university, the purpose of this study was explained. In addition, after promising to provide gift products to students who wish to respond to the survey, cooperation was sought in recruiting research subjects through the person in charge of each university. The purpose and content of the study were fully explained to these participants, and a survey was collected with their consent. A total of 36 surveys that did not have contents filled in fully or gave inconsistent answers were excluded among all survey participants. The general characteristics of the study subjects are shown in Table 1.

### 2.2. LTPA of the Questionnaire

LTPA is cardiovascular and strength-based behavior occurring within recreation, exercise or sport [2]; in this study, the degree of participation in LTPA conducted in leisure time was surveyed. LTPA encompasses walking, climbing, cycling, swimming, tennis, yoga, badminton, aerobics, golf, etc., and the questions were as follows:

‘How long have you been participating in the LTPA?’,

((1) Do not participate in LTPA, (2) Less than 6 months, (3) 6 months~1 year, (4) 1 year~2 years, (5) Over 2 years)

‘How many times a week does you participate in LTPA?’

((1) Do not participate in LTPA, (2) 1 time, (3) 2 times, (4) 3 times, (5) over 4 times)

‘How many hours per week do you participate in LTPA?’

((1) Do not participate in LTPA, (2) Between 0 and 1 h, (3) Between 1 and 2 h, (4) Between 2 and 3 h, (5) More than 3 h). The higher the number, the higher the LTPA participation.

### 2.3. HC of the Questionnaire

HC is the degree of interest in one’s health [27]. It is a tendency to maintain and improve quality of life and well-being as well as one’s own health condition [28]. This study used tools for analyzing the survey questions and differences of individual characteristics of the research subjects. As a survey tool for HC, the questionnaires used in Michaelidou and Hassan, 2007, and Mai and Hoffmann, 2015 (Cronbach’s a 0.890) [29,30] were directly modified by a professor and a researcher to suit this study. There were 6 HC questions in total:

‘I usually care about my health’;

‘I am aware of my health condition’;

‘I feel responsible for my health’;

‘I strive to maintain and promote my health’;

‘I am more interested in my health than anyone else’; 

‘I think the most important thing is health’.

A 5-point Likert scale was used to answer these questions ((1) strongly disagree, (2) disagree, (3) usually, (4) agree, (5) strongly agree). The higher the number, the higher the HC.

### 2.4. Intention to Participate in LTPA of the Questionnaire

Intention to participate in LTPA refers to the willingness to participate in LTPA. The survey tools on intent to participate in LTPA were modified from the items used in Chung and Kang, 2018 (Cronbach’s a 0.915) [31] to suit the present conditions. There were 4 questions in total on intention to participate in LTPA:

‘I will continue to participate in LTPA even if I am busy’;

‘I will continue to participate in LTPA’;

‘I will continue to participate in LTPA even though I am tired’;

‘I will continue to participate in LTPA Even if I am financially difficult’.

A 5-point Likert scale was used to answer these questions ((1) strongly disagree, (2) disagree, (3) usually, (4) agree, (5) strongly agree). The higher the number, the higher the intention to participate in LTPA.

### 2.5. Statistical Analysis

The collected data were analyzed using SPSS 22.0 and AMOS 22 in order to confirm whether the collected questionnaires were suitable for factor analysis. The Kaiser–Meyer–Olkin (KMO) test > 0.5 and the Bartlett test *p* < 0.05 were also used. We performed an exploratory factor analysis to extract common factors for each question. The factor rotation method used Varimax, a right-angle rotation method. Factor extraction was set to an eigenvalue of 1.0 or more and a factor-loading value of 4 or more. We performed confirmatory factor analysis for HC and intent to participate in LTPA for exploratory factor analysis. In order to analyze the validity and reliability of the variables, we measured the fitness by a content validity test. Reliability was analyzed using Cronbach’s α, and discriminant validity was assessed by AVE. We used chi-square, *t*-test, and one-way ANOVA to analyze the differences in HC, amount of LTPA, and intent to participate in LTPA according to the menstruation pattern and the LTPA of Korean university students. Last, to find out the relationship among factors, we carried out Pearson’s correlation coefficient analysis.

### 2.6. Exploratory Factor Analysis

Exploratory factor analysis was performed through the SPSS 22.0 program. Exploratory factor analysis found that KMO was 0.934, which was higher than the standard value of 0.80. The Bartlett test result also showed *p* = 0.000, and the sample was appropriate, lower than the baseline of *p* = 0.05. The Cronbach’s α was more than 0.8 in the reliability of each factor. AVE was also higher than 0.8, which ensured reliability and validity. The exploratory factor analysis results are shown in Table 2.

### 2.7. Confirmatory Factor Analysis

**Figure 1:**
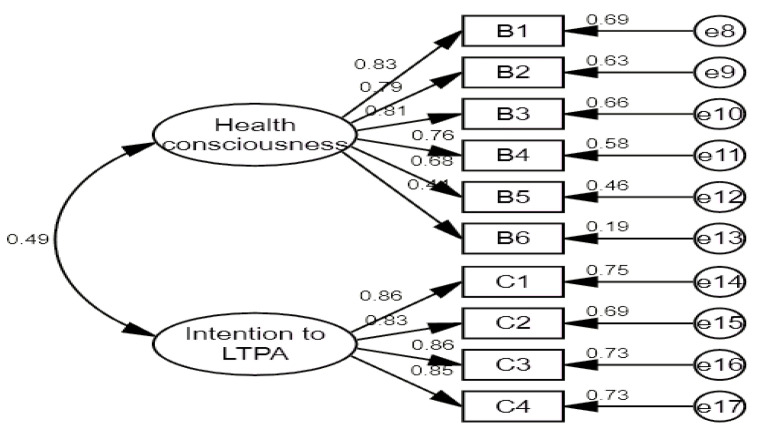
Confirmatory factor analysis showing the suitability of HC and intention to participate in LTPA. Numbers describe the relationship between factors. The reliability values of HC and intention to participate in LTPA are detailed in Table 3.

Exploratory factor analysis was performed through the AMOS 22.0 program. The standardized path coefficients for confirmatory factor analysis are shown in Figure 1.

## 3. Results

### 3.1. Chi-Square among LTPA According to Menstruation

The results of the chi-square test on LTPA according to the menstruation of Korean female university students are shown in Table 4. There were no significant differences in LTPA (χ^2^ = 5.828, *p* < 0.212) according to the menstruation pattern. However, after menstruation ends, female university students with a regular menstruation pattern are shown to have higher LTPA than female university students with irregular menstruations.

### 3.2. t-Test among HC, Amount of LTPA, and Intention to Participate in LTPA by Menstruation

The results of the *t*-test show the differences among HC, amount of LTPA, and intention to participate in LTPA according to the menstruation patterns of Korean female university students (Table 5). First, there was a significant difference between HC (*p* = 0.000) and menstruation. Female university students with regular menstruation (M = 3.732) had higher HC than female students with irregular menstruation patterns (M = 3.416). Second, there was a significant difference between the amount of LTPA (*p* = 0.002) and menstruation pattern. Female university students with regular menstruation (M = 2.829) performed LTPA more than female students with irregular menstruation (M = 2.401). Third, intention to participate in LTPA (*p* = 0.002) and menstruation were significantly different; female university students with regular menstruation (M = 3.243) intended to participate in LTPA more than female university students with irregular menstruation did (M = 2.992).

### 3.3. ANOVA among HC, Amount of LTPA, and Intention to LTPA in LTPA 

The analyzed results of differences among HC, amount of LTPA, and intention to in LTPA according to LTPA of Korean female university students are shown in Table 6. First, in HC, before menstruation (M = 3.745), after menstruation (M = 3.702), and when not menstruating (M = 3.781), LTPA was higher than who did not participate in LTPA (M = 3.338). Second, in amount of LTPA, before menstruation (M = 3.208), after menstruation (M = 3.318), always (M = 3.611) and when not menstruating (M = 3.145), LTPA was higher than do not participate in LTPA (M = 2.780). Third, for intention to participate in LTPA, before menstruation (M = 3.305), after menstruation (M = 3.337), always (M = 3.449) and when not menstruating (M = 3.474), LTPA was higher than do not participate in LTPA (M = 2.581).

### 3.4. Pearson’s Correlation Coefficient Analysis among HC, Amount of LTPA, and Intention to LTPA

Finally, to analyze the relationship among variables, we performed Pearson’s correlation coefficient analysis. As a result, as shown in Table 7, it showed that the relationship among HC, amount of LTPA and intention to participate in LTPA had a positive correlation at the *p* < 0.05 level. The Pearson correlation coefficient showed a positive relationship for HC, amount of LTPA, and intention to participate in LTPA. Amount of LTPA and intention to participate in LTPA were found to have the highest positive relationship. Then, HC and intention to LTP were found to have a positive relationship. Finally, HC and amount of LTPA were found to be high. The higher the HC, the higher the participation in the LTPA, and the higher the intention to continue the LTPA.

## 4. Discussion

This study is to analyze the differences in HC, amount of LTPA, and intention to participate in LTPA according to the menstruation and LTPA of Korean female university students. This study surveyed 500 female university students in three metropolitan cities in Korea using a convenience sampling methodology. The reason was Korea had various limitations due to a lot of confusion and COVID-19. Statistical analysis used chi-square, *t*-test, and one-way ANOVA to analyze the differences in HC, amount of LTPA, and intent to participate in LTPA. Last, to find out the relationship among factors, we carried out Pearson’s correlation coefficient analysis. Therefore, this study’s aim is to understand the leisure time physical activity of female university students in Korea who are experiencing stress due to, e.g., study and employment. Discussions of the results are as follows.

First, there were no significant differences in LTPA and menstruation according to the chi-square among Korean female university students. However, the study showed that female university students with regular menstruation participated in LTPA after menstruation more than female university students with irregular menstruation. In other words, it can be inferred that female university students with irregular menstruation have a lower tendency to participate in LTPA. A study of Korean female university students showed only 51.7–63.0% of female university students had regular menstruations, and more than 30% had irregular menstrual cycles [32,33,34]. As shown from the results of this study, it can be inferred that 30% of female university students with irregular menstruation cycles did not participate in LTPA. Menstrual discomfort occurs in female university students whether they have a regular or an irregular menstrual cycle. As mentioned in the introduction, it is recommended that female university students exercise at least 3 times a week and at least 30 min a day in order to maintain and improve their health, but it will be difficult to perform regular LTPA due to menstruation. However, female college students who have a regular menstruation cycle will be aware of their menstrual cycle and will participate in regular LTPA accordingly. Furthermore, female university students should actively participate in LTPA to maintain regular menstruation.

Second, there were significant differences in the *t*-test among HC, amount of LTPA, and intention to participate in LTPA according to the menstruation of Korean female university students. The participants with regular menstruation showed higher HC, amount of LTPA and intention to participate in LTPA than those with irregular menstruation patterns. A study of women’s health showed that the higher the level of concern for health they have, the more they participate in LTPA [27]. Comparing one’s own body with celebrities, models, and sports stars’ bodies, as mentioned in the introduction, is an unhealthy HC, which could lead to a dangerous diet. This will cause irregular menstruation, which will eventually lead to poor health for female university students in Korea. This study showed that female university students who have regular menstruation cycles participate in LTPA more often and have higher HC. Furthermore, it can be inferred that female university students with regular menstruation cycles benefit in terms of amount of LTPA and intention to participate in LTPA. LTPA positively affects life satisfaction, and the importance of LTPA should be further emphasized for female university students.

Third, there were significant differences in ANOVA among HC, amount of LTPA, and intention to participate in LTPA according to the LTPA of Korean female university students. The HC was higher in those who performed LTPA before menstruation, after menstruation, and when not menstruating than in those who did not participate in LTPA regularly. The amount of LTPA was higher in female university students who participated in LTPA before menstruation, after menstruation, always, and when not menstruating than in those who did not participate in LTPA. The intention to participate in LTPA was higher in ones who participated in LTPA before menstruation, after menstruation, always, and when not menstruating than in those who did not participate in LTPA. In the end, the HC, amount of LTPA, and intention to participate in LTPA were all found to be high among female college students who performed LTPA during periods other than menstruation. In the end, the HC, amount of LTPA, and intention to participate in LTPA were all found to be high among female college students who performed LTPA during periods other than menstruation. In general, estrogen secretion increases gradually during the follicular phase, decreases rapidly during ovulation, and maintains a high level during the luteal phase. Progesterone secretion increases after ovulation and gradually decreases along with estrogen after the middle luteal phase [35]. Morris and Wark [36] stated that stressful situations that may appear during menstruation affect hormone secretion. That is, athletes who participated in long-term exercise showed different hormonal changes according to menstrual cycle from those who did not exercise. Another study found that women with a normal menstrual cycle had higher motor performance in the late follicular phase than in the middle luteal phase. Estrogen is said to constrict blood vessels and improve the working capacity of the heart [37]. The results of this study show that female university students participate in more LTPA after menstruation, which is psychologically and physically more comfortable than before menstruation. After all, students who have high LTPA levels tend to be interested in their own health. Furthermore, it can be inferred that the amount of LTPA and the intent to participate in LTPA are related. Women who participate in LTPA during menstruation have fewer menstrual pains than women who do not participate in LTPA [38,39]. Women’s menstruation often occurs when physiological hormonal changes occur and LTPA is reduced due to more fatigue than usual. However, even during this time, the habit of LTPA can help women feel better, and boost energy in the body. Regular LPTA participation helps you enjoy various benefits. Individuals who are inactive due to menstruation not only lose physical and psychological benefits, but are also more likely to be exposed to various diseases. In particular, considering the rhythm of hormones in women, it would be more effective to participate in LTPA in the morning before energy levels drop [40]. Therefore, it is suggested that Korean female university students should always participate in LTPA for long-term health benefits.

## 5. Conclusions

In this study, female university students showed different HC, amount of LTPA, and intention to participate in LTPA according to menstruation cycle regularity (regular, irregular). In other words, it was confirmed that women’s awareness of menstruation and concerns about irregular menstruation decreased LTPA. Therefore, female university students ultimately have different HC and participation levels in LTPA depending on menstruation pattern, and it is necessary to improve these perceptions or reconsider health awareness in terms of levels suitable for different menstruation patterns. This study was conducted at a time when COVID-19 had pandemic Korea. Therefore, due to many restrictions on recruiting research subjects, convenient sampling methods were used. In future studies, it will be necessary to secure the reliability of the study through probability sampling. This study is a preliminary study to analyze the relationship between menstrual cycle and LTPA of Korean female university students. However, there were restrictions on the selection of study subjects and participation in the LTPA due to COVID-19. Therefore, in the next study, a more extensive selection should be made in the selection of subjects, and a wide-ranging level of research should be conducted in LTPA participation.

## Figures and Tables

**Table 1:** The individual characteristics of the study subjects (N = 464).

Variables			N (464)	%
Menstruation		Regular	288	62.1
		Irregular	176	37.9
Age		19s	29	6.3
		20s	161	34.7
		21s	109	23.5
		22s	94	20.3
		Over 23s	71	15.2
LTPA		Do not participate in LTPA	129	27.8
		Before menstruation	99	21.3
		After menstruation	134	28.9
		Always	54	11.6
		When not menstruating	48	10.3
Amount of LTPA	Exercise duration	Do not participate in LTPA	129	27.8
		Less than 6 months	141	30.4
		6 months~1 year	50	10.8
		1 year~2 years	39	8.4
		Over 2 years	105	22.6
	Exercise frequency (1 week)	Do not participate in LTPA	129	27.8
		1 time	80	17.2
		2 times	95	20.5
		3 times	87	18.8
		over 4 times	73	15.7
	Exercise Time (1 day)	Do not participate in LTPA	129	27.8
		Between 0 and 1 h	86	18.5
		Between 1 and 2 h	156	33.6
		Between 2 and 3 h	50	10.8
		More than 3 h	43	9.3

**Table 2:** Validity and reliability analysis.

Variable	Questionnaire	Factor Loading	Eigen Value	Cronbach’s α	AVE
HC	B1, I usually care about my health	0.847	3.977	0.864	0.943
	B2, I am aware of my health condition	0.812			
	B4, I feel responsible for my health	0.765			
	B3, I strive to maintain and promote my health	0.737			
	B5, I am more interested in my health than anyone else	0.644			
	B6, I think the most important thing is health	0.432			
Intention to LTPA	C4, I will continue to participate in exercise Even if I am financially difficult	0.875	1.817	0.912	0.970
	C3, I will continue to participate in exercise even though I am tired	0.820			
	C1, I will continue to participate in exercise even if I am busy	0.715			
	C2, I will continue to participate in the exercise	0.696			

((1) strongly disagree, (2) disagree, (3) usually, (4) agree, (5) strongly agree). The higher the number, the higher the HC. KMO = 0.924; Bartlett test = 6117.178; df = 190; *p* = 0.000. Matrix of correlation coefficients used in factor analysis. Factor loading: A value indicating the correlation between factors and variables in factor analysis. Eigenvalues: Extracted sum of squares loading. Cronbach’s α: Reliability Index.

**Table 3:** Confirmatory factor analysis of HC and intention to participate in LTPA.

Path	Non-Standard Estimate	Standard Estimate	S.E.	C.R.
B1 ← HC	1.000	0.833	-	-
B2 ← HC	0.894	0.794	0.046	19.249 ***
B3 ← HC	1.048	0.811	0.053	19.815 ***
B4 ← HC	0.865	0.760	0.048	18.151 ***
B5 ← HC	0.967	0.681	0.061	15.744 ***
B6 ← HC	0.499	0.439	0.053	9.104 ***
C1 ← Intention to participate in LTPA	1.000	0.863	-	-
C2 ← Intention to participate in LTPA	0.863	0.831	0.039	22.351 ***
C3 ← Intention to participate in LTPA	1.021	0.856	0.044	23.455 ***
C4 ← Intention to participate in LTPA	1.027	0.854	0.044	23.371 ***

((1) strongly disagree, (2) disagree, (3) usually, (4) agree, (5) strongly agree). The higher the number, the higher the HC, *** *p* < 0.001. Estimate is a non-standardized value, S.E. (Standard error), and the C.R. (Critical ratio) value must be 1.96 or more.

**Table 4:** Chi-square among LTPA according to menstruation.

Variable	Menstruation			
	Regular (N, %)	Irregular (N, %)	Total (N)	χ^2^ (*p*)
LTPA	288 (62.1)	176 (37.9)	464	5.828 (0.212)
Do not participate in LTPA	69 (53.5)	60 (46.5)	129	
Before menstruation	63 (63.6)	36 (36.4)	99	
After menstruation	89 (66.4)	45 (33.6)	134	
Always	36 (66.7)	18 (33.3)	54	
When not menstruating	31 (64.6)	17 (35.4)	48	

((1) strongly disagree, (2) disagree, (3) usually, (4) agree, (5) strongly agree). The higher the number, the higher the HC. Menstruation: bleeds regularly on average every 28 days, and irregular means does not. LTPA: Do not participate in LTPA, before menstruation (luteal phase), after menstruation (period excluding menstruation in the follicular phase), when not menstruating (any period without menstruation phase), always. N: Number of LTPA responses by menstruation. %: Ratio to the number. χ^2^: chi-square. *p*: significance level.

**Table 5:** *t*-test among HC, amount of LTPA, and intention to participate in LTPA by regular or irregular menstruation.

Variable	Regular		Irregular		t	*p*
	M	SD	M	SD		
HC	3.732	0.6478	3.416	0.000	5.122 ***	0.000
Amount of LTPA	2.829	1.242	2.401	0.002	3.659 ***	0.002
Intention to participate in LTPA	3.243	0.8639	2.992	0.002	3.092 ***	0.002

((1) strongly disagree, (2) disagree, (3) usually, (4) agree, (5) strongly agree), The higher the number, the higher the HC. *** *p* < 0.001; M: Average of the total score of the questions answered; SD: Standard deviation; *t*: Value of *t*-test; *p*: significance level.

**Table 6:** ANOVA among HC, amount of LTPA, and intention to participate in LTPA.

Variable	LTPA	N	M	(SD)	F	*p*
HC	Do not participate in LTPA	129	3.338	0.6332	8.478	0.000 ***
	Before menstruation	99	3.745	0.6363		
	After menstruation	134	3.702	0.5646		
	Always	54	3.642	0.6740		
	When not menstruating	48	3.781	0.8158		
Amount of LTPA	Do not participate in LTPA	129	2.374	0.7453	14.001	0.000 ***
	Before menstruation	99	2.925	0.8060		
	After menstruation	134	3.010	0.7957		
	Always	54	2.827	0.8824		
	When not menstruating	48	3.104	0.7844		
Intention to participate in LTPA	Do not exercise	129	2.581	0.8023	23.854	0.000 ***
	Before menstruation	99	3.305	0.7656		
	After menstruation	134	3.337	0.7178		
	Always	54	3.449	0.8261		
	When not menstruating	48	3.474	0.8965		

((1) strongly disagree, (2) disagree, (3) usually, (4) agree, (5) strongly agree), The higher the number, the higher the HC. *** *p* < 0.001; N: Number of LTPA responses according to HC, Amount of LTPA, Intention to LTPA; M: Average of the total score of the questions answered; SD: Standard deviation; F: Value of ANOVA; *p*: significance level.

**Table 7:** Pearson’s correlation coefficient analysis among HC, amount of LTPA, and intention to participate in LTPA.

Variables	HC	Amount of LTPA	Intention to Participate in LTPA
HC	1	-	-
Amount of LTPA	0.274 **	1	-
Intention to participate in LTPA	0.448 **	0.468 **	1

((1) strongly disagree, (2) disagree, (3) usually, (4) agree, (5) strongly agree). The higher the number, the higher the HC. ** *p* < 0.01. The Pearson correlation coefficient showed a positive relationship for HC, amount of LTPA, and intention to participate in LTPA.
